# Biodentine™ Partial Pulpotomy of a Young Permanent Molar with Signs and Symptoms Indicative of Irreversible Pulpitis and Periapical Lesion: A Case Report of a Five-Year Follow-Up

**DOI:** 10.1155/2019/8153250

**Published:** 2019-09-12

**Authors:** W. Chinadet, T. Sutharaphan, P. Chompu-inwai

**Affiliations:** ^1^Division of Pediatric Dentistry, Department of Orthodontics and Pediatric Dentistry, School of Dentistry, Chiang Mai University, Thailand; ^2^Phrae Hospital, Phrae, Thailand

## Abstract

The purpose of this paper was to report the five-year success of Biodentine™ partial pulpotomy in a young permanent molar, with signs and symptoms indicative of irreversible pulpitis and periapical lesion, in a nine-year-old girl. Preoperative clinical examination revealed a large carious lesion of the left mandibular permanent first molar. The patient reported pain on percussion. The tooth responded positively to the electric pulp test and had lingering pain after cold testing. A periapical radiograph showed a deep carious lesion and periapical lesion. Based on the clinical and radiographical examination, the tooth had signs and symptoms indicative of irreversible pulpitis and periapical lesion. During caries removal, pulp exposure occurred, and 2-3 mm in depth of pulp tissue at the exposure site was removed. Haemorrhage was controlled within four minutes with 2.5% sodium hypochlorite-moistened cotton pellets. Biodentine™ was then applied as both a pulp dressing and a temporary restoration. At the following visit, composite resin was placed over the Biodentine™ as a final restoration. During a five-year follow-up, the tooth was asymptomatic, had positive responses to sensibility tests, and had no discolouration. Follow-up radiographs showed a dentine bridge and periapical healing.

## 1. Introduction

Partial pulpotomy, one form of vital pulp therapy, has traditionally been indicated only in a cariously or traumatically exposed vital tooth with a clinical diagnosis of normal pulp or reversible pulpitis [1]. When a tooth has signs and symptoms indicative of irreversible pulpitis and periapical lesion, root canal treatment has traditionally been recommended [1]. However, root canal treatment is an expensive, complicated, and time-consuming procedure. Recently, the success of vital pulp therapy in challenging cases has been demonstrated [2].

Mineral trioxide aggregate (MTA) has been recommended as the gold standard for vital pulp therapy; however, it has some disadvantages, such as long setting time, poor handling properties, high cost, and the potential for tooth discolouration [3]. Biodentine™ (Septodont, Saint-Maur-des-Fossés, France), one of the new-generation, bioactive endodontic cements, has been claimed to have improved properties over MTA.

Biodentine™ presents high biocompatibility with human dental pulp cells [4]. The pulp tissue in contact with Biodentine™ does not show an irreversible inflammatory response [5]. Previous studies have reported that Biodentine™ has high antibacterial effects and antifungal activity [6, 7]. It has a shorter setting time than MTA, as the result of calcium chloride in the liquid component of Biodentine™ [8]. The easy manipulation of Biodentine™ is the result of hydrosoluble polymer in the liquid component, which is based on polycarboxylate, a component that induces flocculation and increases flowability at low water/cement ratios [9]. Biodentine™ has higher flexural strength [10], compressive strength [11], and modulus of elasticity than does MTA [12]. Consequently, Biodentine™ can be used simultaneously as a pulp dressing as well as a base material. Moreover, bismuth oxide in MTA is replaced with zirconium oxide in Biodentine™, thus resulting in better colour stability [13].

The purpose of this paper was to report the long-term success of partial pulpotomy using Biodentine™ in a young permanent molar, with signs and symptoms indicative of irreversible pulpitis and periapical lesion.

## 2. Case Report

**Figure 1:**
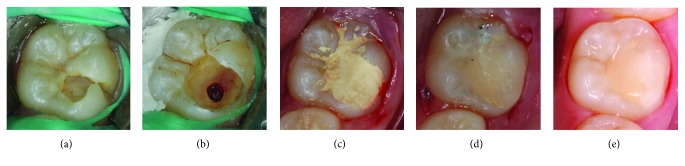
Intraoral photographs: (a) initial clinical appearance, (b) after pulp tissue removal and haemorrhage was controlled, (c) Biodentine™ was placed as a temporary filling, (d) restored with composite resin, and (e) colour stability of the tooth after a five-year follow-up.

**Figure 2:**
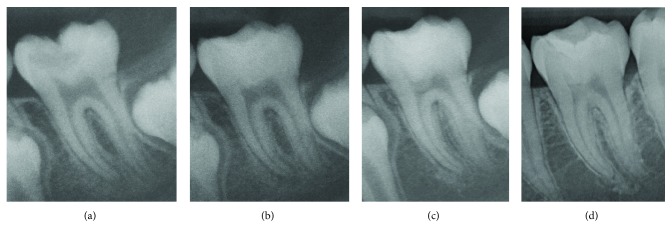
Periapical radiographs recorded (a) before treatment, (b) immediately after treatment, (c) six months postoperative, and (d) five years postoperative, showing dentine bridge and improvement of the periapical area.

A nine-year-old healthy Thai girl presented to the Pediatric Dentistry Clinic, Faculty of Dentistry, Chiang Mai University, because of pain in the lower left quadrant upon eating and upon drinking cold liquids. Clinical examination revealed a large carious lesion involving the occlusal and buccal surfaces of the left mandibular permanent first molar (Figure 1(a)). The patient reported pain on percussion. The tooth responded positively to the electric pulp test (EPT; Kerr Vitality Scanner; SybronEndo, Glendora, CA, USA) and had lingering pain for more than 10 seconds after cold testing (Green Endo-Ice®, Coltene Whaledent, Cuyahoga Falls, OH, USA). A periapical radiograph showed a deep carious lesion in close proximity to the pulp and periapical lesion, including loss of lamina dura, widened periodontal ligament space, and condensing osteitis (Figure 2(a)). Based on the clinical and radiographical examination, the tooth had signs and symptoms indicative of irreversible pulpitis and periapical lesion. The options of treatment, root canal treatment or vital pulp therapy, with their risks and benefits were explained to the patient and her legal guardian. Both preferred vital pulp therapy and signed assent and informed consent forms, respectively.

A local anaesthetic was administered, and a rubber dam was placed. During caries removal, there was a carious pulp exposure, approximately 2.5 mm in diameter. The exposed pulp appeared vital, judged by its appearance, bright red colour, and bleeding overflowing from the exposure site. Then, approximately 2-3 mm in depth of pulp tissue at the exposure site was removed with a sterilized long-shank, high-speed, round carbide bur and irrigated with 2.5% sodium hypochlorite (NaOCl). The bleeding was controlled with 2.5% NaOCl-moistened cotton pellets within four minutes (Figure 1(b)). Biodentine™ was mixed according to the manufacturer's instructions and then applied to the exposed site and surrounding dentine as a pulp dressing as well as a temporary restorative material. Biodentine™ was allowed to initially set for 12 minutes (Figure 1(c)). A periapical radiograph was recorded immediately after treatment (Figure 2(b)).

**Figure 3:**
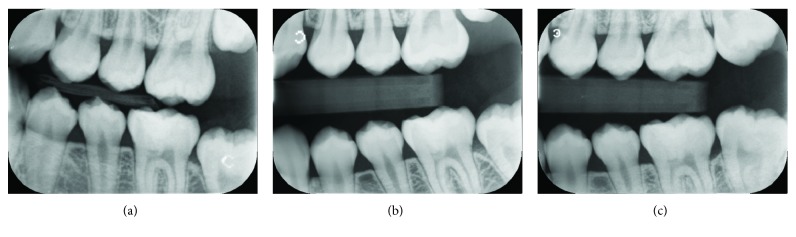
Bitewing radiographs recorded (a) three years postoperative, (b) four years postoperative, and (c) five years postoperative, showing dentine bridge in the left mandibular permanent first molar.

Two days later, the patient returned for a definitive restoration. The outer layer of Biodentine™ was removed leaving 2 mm of space for final restoration with resin composite. A two-step total-etch adhesive (Scotchbond™ etchant and Adper™ Single Bond; 3M ESPE, St. Paul, MN, USA) was applied, according to the manufacturer's instructions, over the Biodentine™ and the surrounding dentine wall. Finally, the cavity was filled with resin composite (Filtek™ Z350 XT; 3M ESPE) and light cured for 40 seconds for each surface, followed by the finishing and polishing procedures (Figure 1(d)). The treated tooth was followed up every six months, for five years. The tooth had positive responses to EPT and cold testing. The colour stability of the tooth was noted (Figure 1(e)). The follow-up periapical radiographs showed a dentine bridge and periapical healing (Figures 2(c) and 2(d)). Bitewing radiographs at different follow-up periods showed a dentine bridge underneath the Biodentine™ layer (Figure 3).

## 3. Discussion

Although contrary to the traditional recommendation [1], partial pulpotomy was performed successfully in this case with signs and symptoms indicative of irreversible pulpitis and periapical lesion. Clinical diagnosis of the pulp status has traditionally been used as the main criterion for choosing treatment; however, the correlation between clinical and histological pulpal status varies from weak to high [14, 15]. Moreover, a periapical lesion in teeth with vital pulp in young patients may be the result of the process of immunological response to an irritating factor invading from the corona, diffusing through the radicular pulp tissue [16]. Information derived from preoperative clinical and radiographic examination should not be the sole criterion used to determine treatment, and direct evaluation of the pulp can also aid in the evaluation of pulp vitality.

When there is a pulp exposure, the appearance of the pulp tissue, the colour of the bleeding, and haemorrhage control at the exposure site are also clinical criteria commonly used to judge the vitality of the pulp. In this case report, the exposed pulp appeared vital, judged by its resilient texture, bright red colour, and bleeding overflowing from the exposure site. After pulp amputation, the bleeding was controlled within four minutes, indicating that the remaining pulp tissue was healthy and thus could be preserved. Some authors agree that pulpal bleeding can be used as a clinical indicator of pulpal inflammation [16, 17]; however, there is no consensus regarding the specific time required to control bleeding. On the other hand, Mutluay et al. [18] suggested that the evaluation of pulpal bleeding is subjective and may not reflect the actual pulpal status. Methods aiding in correct diagnosis of the pulp status should be further studied.

Moreover, there is still controversy regarding the type of irrigating solution and haemostatic agent most suitable for vital pulp therapy. NaOCl has been one of the most commonly used disinfectants for root canal treatment for many years; consequently, clinicians have arbitrarily adopted the use of NaOCl for vital pulp therapy [17, 19, 20]. NaOCl readily controls bleeding, while at the same time disinfects the cavity [19]. One point five per cent to 6% NaOCl appears to be the most effective and inexpensive haemostatic agent for direct pulp capping procedures [20, 21] because these concentrations do not negatively affect pulp cell recruitment, cytodifferentiation, or reparative dentin deposition when used against pulp tissue [22]. Moreover, NaOCl has been recommended as a diagnostic tool to assess inflammation of pulpal tissue; when haemorrhage is controlled within ten minutes, the prognosis can be favorable [23].

Similar to our case report, previous studies have reported successful outcomes of vital pulp therapy, using bioactive endodontic cement, in teeth diagnosed with irreversible pulpitis and periapical lesion [17, 24]. The advancement of bioactive endodontic cements may play a role in the success of challenging cases. The follow-up radiographs of the presented case show a dentine bridge underneath the Biodentine™ layer. The odontogenic effect of Biodentine™ involves the creation of a suitable environment for healing, including the release of calcium ions, production of calcium hydroxide, and formation of an apatite-like layer between the contact surface of dentine and the material [3, 4]. Moreover, Biodentine™ has been shown to stimulate the release of bioactive molecules, such as transforming growth factor-*β*1 (TGF-*β*1), nerve growth factor (NGF), and glial cell line-derived growth factor (GDNF) in dentine, contributing to the induction of tertiary dentinogenesis [25]. De Rossi et al. evaluated the pulpal and periapical responses of dogs' teeth after pulpotomy with Biodentine™ and MTA and demonstrated that Biodentine™ allows for mineralized tissue bridge formation after pulpotomy in all specimens with similar morphology and integrity to those formed with use of MTA [26].

Compared to MTA, Biodentine™ has a shorter setting time, better physical and mechanical properties, and easier handling [27]. The sealing ability of Biodentine™ has been shown to be equal to or better than that of MTA [28], thus allowing it to be used as a temporary restorative material. Moreover, as shown in this case report, Biodentine™ does not cause discolouration of the treated tooth because it contains zirconium dioxide, instead of bismuth oxide, the radiopacifier that causes tooth discolouration when MTA is used [29].

In conclusion, Biodentine™ might be a suitable biomaterial in partial pulpotomy of young permanent molars, with signs and symptoms indicative of irreversible pulpitis and periapical lesion. However, further clinical studies with longer follow-up periods and larger sample sizes are recommended.
